# Clinical Effect of Surgical Correction for Nasal Pathology on the Treatment of Obstructive Sleep Apnea Syndrome

**DOI:** 10.1371/journal.pone.0098765

**Published:** 2014-06-04

**Authors:** Chong Yoon Park, Joon Hyeong Hong, Jae Heon Lee, Kyu Eun Lee, Hyun Sang Cho, Su Jin Lim, Jin Wook Kwak, Kyung Soo Kim, Hyun Jik Kim

**Affiliations:** Department of Otorhinolaryngology and Head & Neck Surgery, Chung-Ang University College of Medicine, Seoul, Korea; Beijing Institiute of Otolaryngology, China

## Abstract

**Objectives:**

This study aimed to evaluate the hypothesis that relief of nasal obstruction in subjects with obstructive sleep apnea (OSA) would lead to reduce OSA severity and to discuss the available evidence on the clinical efficacy of nasal surgery as a treatment modality for OSA.

**Study Design:**

Twenty-five subjects who had reduced patency of nasal cavity and narrowing of retroglossal or retropalatal airways were diagnosed with OSA and underwent nasal surgery, such as septoplasty or turbinoplasty to correct nasal pathologies. The effect of the surgery on nasal patency was quantified by measuring minimal cross-sectional area (MCA) using acoustic rhinometry. The watch-PAT-derived respiratory disturbance index (RDI), apnea and hypopnea index (AHI), lowest oxygen saturation, and valid sleep time were measured before and after nasal surgery.

**Results:**

The present study shows that the AHI and RDI decreased significantly and the lowest oxygen saturation and valid sleep time rose after nasal surgery in 25 OSA subjects. In addition, a reduction in subjective symptoms was observed in subjects and mean MCA increased after nasal surgery. Fourteen subjects were classified as responders and 11 subjects as non-responders. Responders showed considerable improvement of their subjective symptoms and the AHI and RDI were significantly lower after surgery. We found that the changes between pre- and post-operative AHI and RDI values were minimal in 11 non-responders. However, daytime somnolence and REM sleep time improved after nasal surgery in non-responders.

**Conclusions:**

Our study provides evidence that the surgical treatment of nasal pathology improves nasal airway patency and reduces OSA severity in 56% subjects. Furthermore, correction of nasal pathology appears to result in improved sleep quality in both responder and non-responders OSA subjects.

## Introduction

Obstructive sleep apnea (OSA) is a common sleep disorder and is characterized by airway collapse at multiple levels of upper airway, which cause reduction or cessation of airflow. It may cause nighttime hypoxemia or vascular injury due to free oxygen radicals and even lead to cardiovascular, endocrinologic and neurocognitive diseases without proper diagnosis treatment [1], [2]. Nasal obstruction is known to contribute to OSA since the nasal cavity contributes one-half to two-thirds of total airway resistance [3]. Under normal circumstance, breathing during sleep is primarily nasal rather than oral and considerable evidences suggest that resolution of nasal obstruction improves OSA severity [4]. In the Starling resistor model, wherein the upper airway is described as a hollow tube, nasal obstruction increases negative pressure in the pharyngeal airway and predisposes to pharyngeal collapse as in a collapsible down-stream segment [5]. It has been reported that airflow resistance of the nasal cavity is significantly higher in patients with OSA compared to patients with simple snoring and this fact indicates that correction of nasal obstruction may be an important treatment modality for patients with OSA [6]. Different therapeutic options have been proposed by researchers to improve nasal breathing of OSA patients, including medical treatments and surgical interventions [7]–[9]. Medical treatments for nasal obstruction have been studied for conditions such as snoring, upper airway resistance syndrome, and OSA with allergic rhinitis. Nasal decongestants have been known to reduce snoring frequency and intensity in OSA patients with nasal obstruction [10]. Similarly, it has been reported that topical nasal steroid use in patients with allergic rhinitis is associated with improved subjective sleep quality and apnea-hypopnea index (AHI) in adults [11]. Nasal surgery has also been used to treat OSA. The absence of pharyngeal narrowing in patients with OSA differentiates those with a better response to nasal surgery [12]. Surgery for correcting obstructive nasal pathologies such as septal deviation and turbinate hypertrophy could improve sleep apnea. Surgery is also indicated for patients who cannot tolerate CPAP, who don't have a considerable reduction of their AHI using CPAP alone, or who may not be able to use a CPAP machine because of their employment [13], [14].

Despite these well-documented associations, the impact of treatment for nasal pathology on OSA severity is inconsistent due to lack of adequate objective outcome measurements which make the clinical results rather confusing. Therefore, the therapeutic effect of improving nasal airway patency on OSA severity is highly variable and the objective benefits of mechanical improvement for nasal breathing still remain controversial.

In the present study, our purpose was to evaluate if surgical correction of nasal obstruction would reduce OSA severity in subjects with OSA by performing a pre- and postoperative sleep study using the watch-PAT device. We also sought to investigate whether surgical correction of nasal pathology could improve sleep quality and auto-titrating positive airway pressure (autoPAP) adherence.

## Methods

### Ethics statement

Fifty-five adult subjects who had been diagnosed with OSA at Chung-Ang University Hospital (Seoul, Korea) from March 2011 to February 2013 participated in the study. All subjects participated in the study voluntarily and the medical records of the participants were reviewed retrospectively. Written informed consent was obtained from each participant and the study complied with the Declaration of Helsinki. The institutional board of Chung-Ang University Hospital approved this study.

### Subjects

**Figure 1:**
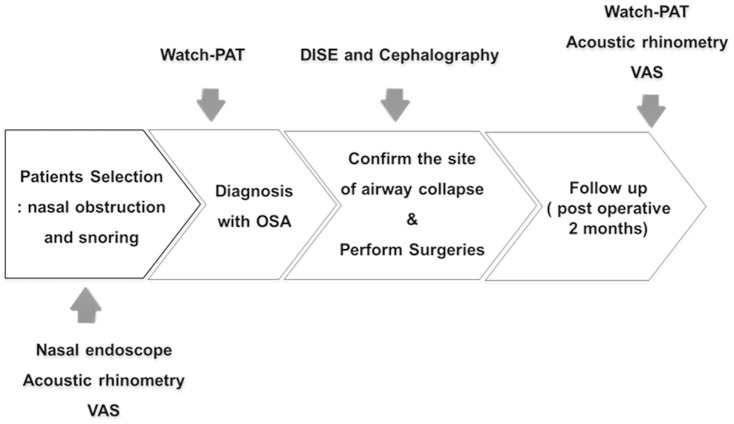
Diagnostic approach to OSA and post-operative evaluation of study subjects. VAS: Visual analogue scale, DISE: Drug-induced sleep endoscopy, OSA: Obstructive sleep apnea.

Fifty five subjects complaining of nasal obstruction and snoring, who has been diagnosed with mild, moderate or severe OSA without tonsil hypertrophy. The watch-PAT 200 (Itamar Medical Ltd, Caesarea, Israel) was used for the diagnosis of OSA and assessment of OSA severity. Cephalography and drug-induced sleep endoscopy (DISE) were performed to evaluate airway narrowing and the anatomical site of airway collapse 1 month before surgery. Intranasal endoscopy and acoustic rhinometry were performed both at 1 month prior to and 2 month post-surgery to estimate nasal airflow and upper airway narrowing (Figure 1). Nasal septal deviation and inferior turbinate hypertrophy were observed in all 55 subjects and the subjects who also showed airway narrowing in the retropalatal or retroglossal area were included in this study. For primary treatment, 25 subjects received septoplasty and turbinoplasty to improve nasal airway patency; correction of retropalatal or retroglossal area narrowing was not carried out in these subjects because symptomatic nasal obstruction was more prominent feature and a more dominant aspect of the OSA pathophysiology in those patients. These 25 subjects were included in the present study to evaluate the influence of nasal surgeries on OSA severity and sleep quality. Subjects with a history of peripheral vasculopathy or autonomic nervous system dysfunction, cardiac or lung disease, use of alpha-adrenergic receptor-blocking agents, and finger deformity that may affect application of the watch-PAT probe were excluded from the study. The patient population consisted of 23 males and 2 females. The mean body mass index was 21.3 kg/m^2^ and the mean age was 47.4 years.

### AutoPAP therapy and therapeutic pressure

AutoPAP therapy was initiated in the outpatient clinic setting as part of routine clinical care. During the first visit, patient education regarding OSA and complications and treatment benefits, nasal mask fitting and device education were accomplished. The optimal therapeutic pressure for the autoPAP (ResMed S9™) was set ranged from 5 to 15 cmH2O with a mean setting of 10.2 cm H2O. Following one week of autoPAP therapy, the subjects' adherence, pressure delivered, air leak levels, and residual events of OSA were examined. Adequate adherence during autoPAP therapy was strictly defined as device usage >4 h/night every nights during the first week.

### Study design and sleep study

All subjects were required to complete a pre- and post-operative research survey that included a visual analogue scale (VAS, scale 1–10 where 10 was completely satisfied) for nasal obstruction, snoring and apnea. An Epworth sleepiness scale (ESS) was used to evaluate subjects' daytime sleepiness. Acoustic rhinometry was performed twice: 1 month prior to the nasal surgeries and 2 months after surgery to examine differences in the nasal minimal cross-sectional area (MCA). The watch-PAT was also performed 1 month before and 2 month after surgeries and pAHI, Respiratory Distress Index (pRDI), lowest oxygen saturation, and valid sleep time were evaluated (Figure 1). We assessed the degree of apnea through AHI and used RDI to determine the severity of OSA or to define responders or non-responders to nasal surgeries. To analyze the watch-PAT results, PAT studies were uploaded for automated analysis on a personal computer using the COMPACTFLASH reader provided with the PAT software (zzz_PAT version 1.5.44.7, Itamar Medical Ltd, Caesarea, Israel).

### Statistical analysis

Kendall tau-b was used to assess correlation and agreement between the surgical results and the watch-PAT data. All analysis was performed with SPSS (version 18.0; SPSS Inc., Chicago, IL, USA) for Windows software. A *p* value <0.05 was considered statistically significant.

## Results

### Clinical characteristics

**Figure 2:**
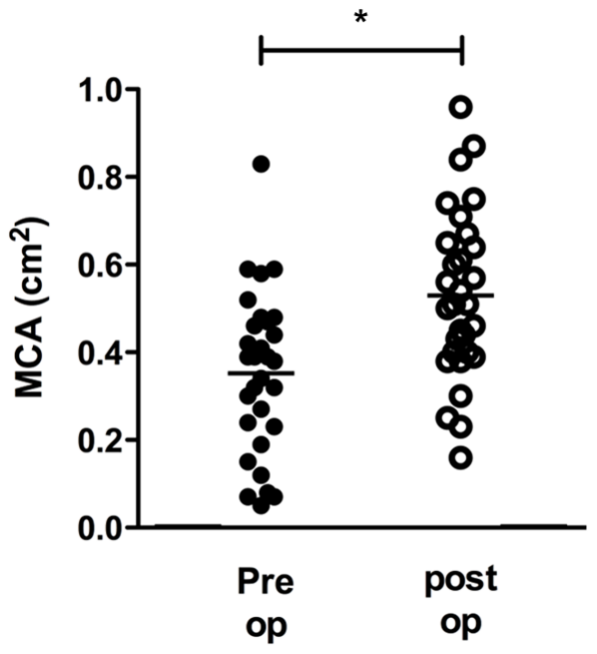
Changes in mean cross sectional area (MCA, cm^2^), as measured by acoustic rhinometry, pre- and post-nasal surgery. ^*^: p<0.05 when comparing the pre-and post-operative values.

Twenty-five OSA subjects who had narrow patency of nasal cavity and narrowing of retroglossal or retropalatal airways were recruited. Septoplasty and turbinoplasty were performed on all subjects as primary treatment. After two months, there was improvement in subjective symptoms and intranasal endoscopy revealed that the nasal airway had widened and nasal obstruction had significantly improved in all subjects. Acoustic rhinometry results demonstrated that septoplasty with turbinoplasty proved to be successful in improving nasal patency with an MCA increase from 0.34±0.2 cm^2^ to 0.58±0.2 cm^2^ after surgery (*p*<0.05) (Figure. 2).

### The analysis of sleep parameters and subjective symptoms after nasal surgeries on OSA subjects

**Figure 3:**
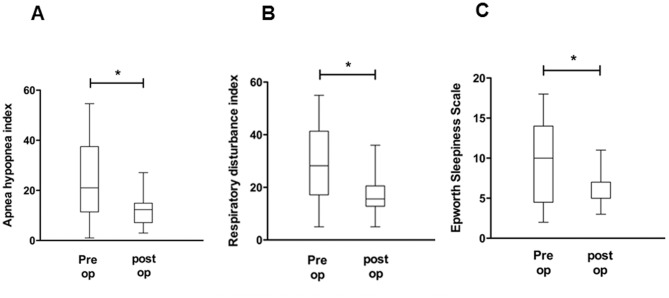
Changes in watch-PAT-derived factors, such as AHI (A), RDI (B), and ESS score (C), before and after nasal surgery. ^*^: p<0.05 when comparing the pre-and post-operative values.

**Figure 4:**
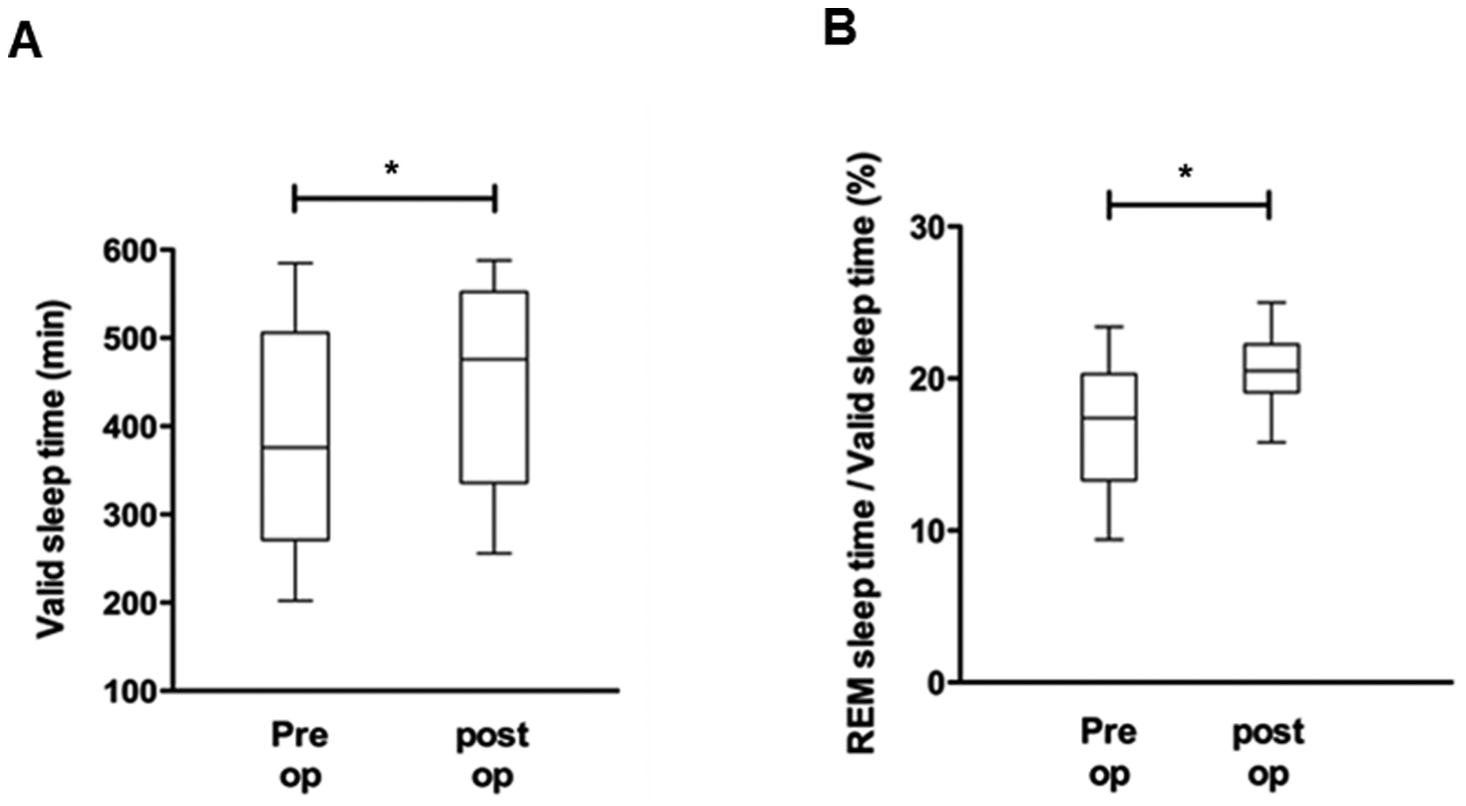
Changes in watch-PAT-derived factors, such as valid sleep time (A) and percentage of REM sleep (B), before and after nasal surgery. ^*^: p<0.05 when comparing the pre-and post-operative values.

As a next step, pre- and post-operative pAHI, pRDI, ESS, valid sleep time (minutes), and REM sleep time (%) were compared to evaluate the effect of nasal surgeries on OSA improvement and advancement of sleep quality. The mean pAHI for the 25 subjects was 23.9±14.9 events per hour when measured before septoplasty with turbinoplasty using the watch-PAT, while the post-operative mean pAHI was 12.2±6.4 events per hour, a statistically significant reduction of pAHI after nasal surgeries (*p*<0.05) (Figure. 3A). The pre-operative mean pRDI was 28.8±14.4 events per hour, while the post-operative mean pRDI was 17.1±7.5 events per hour; thus, post-operative pRDI also decreased considerably after surgical correction (*p*<0.05) (Figure. 3B). The mean value of preoperative ESS to assess patients' daytime sleepiness was 9.68±4.7, which declined to 5.84±2.1 after nasal surgeries (Figure. 3C). The pre-operative valid sleep time was 399.9±122.7 minutes, while the post-operative value was 459.4±110.4 minutes (*p*<0.05) (Figure. 4A). There was also significant improvement in the percentage of REM sleep time with a pre-operation value of 16.6±4.3% and a post-operation value of 20.6±2.0% (*p*<0.05) (Figure. 4B). These results show that surgical correction of nasal obstruction is relatively effective at reducing OSA severity, as demonstrated by improvement in the pAHI, pRDI, valid sleep time, and REM sleep time values using the watch-PAT. Furthermore, daytime sleepiness of subjects also decreased after nasal surgery.

**Figure 5:**
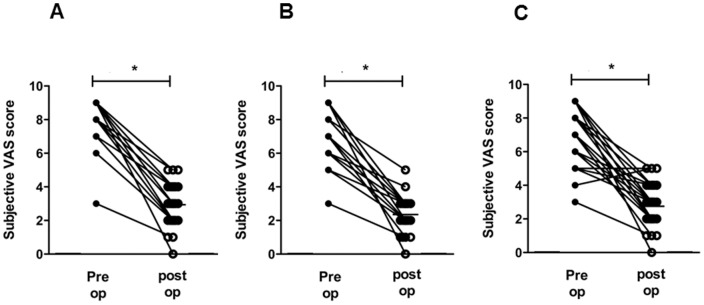
Changes in visual analog scale (VAS) score for subjective symptoms, such as nasal obstruction (A), snoring (B), and daytime somnolence (C), before and after nasal surgery. ^*^: p<0.05 when comparing the pre-and post-operative values.

We assessed the subjective symptom scores for OSA using the VAS score, which evaluates nasal obstruction, snoring, and apnea. Using the VAS scores, nasal obstruction improved from 8.2±0.4 to 3.2±0.1 after surgery, snoring improved from 6.6±1.0 to 2.4±0.7, and apnea improved from 7.2±1.5 to 2.9±1.1 with each improvement being statistically significant (*p*<0.05) (Figure. 5A, 5B, 5C).

### The clinical impact of nasal surgeries on OSA symptoms and autoPAP therapy in non-responder group

**Figure 6:**
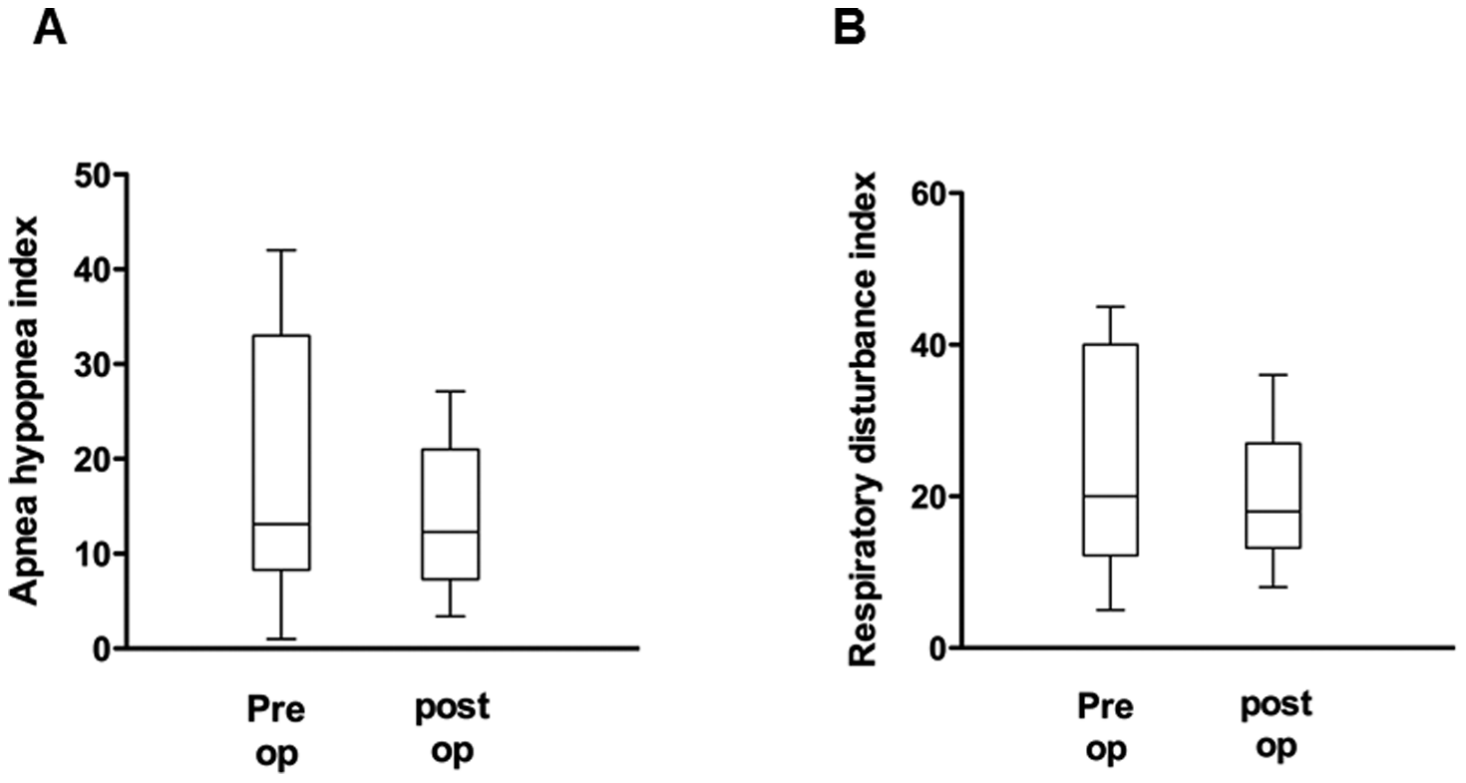
Changes in watch-PAT-derived factors, such as AHI (A) and RDI (B), before and after nasal surgery in the non-responder group (N = 11).

**Figure 7:**
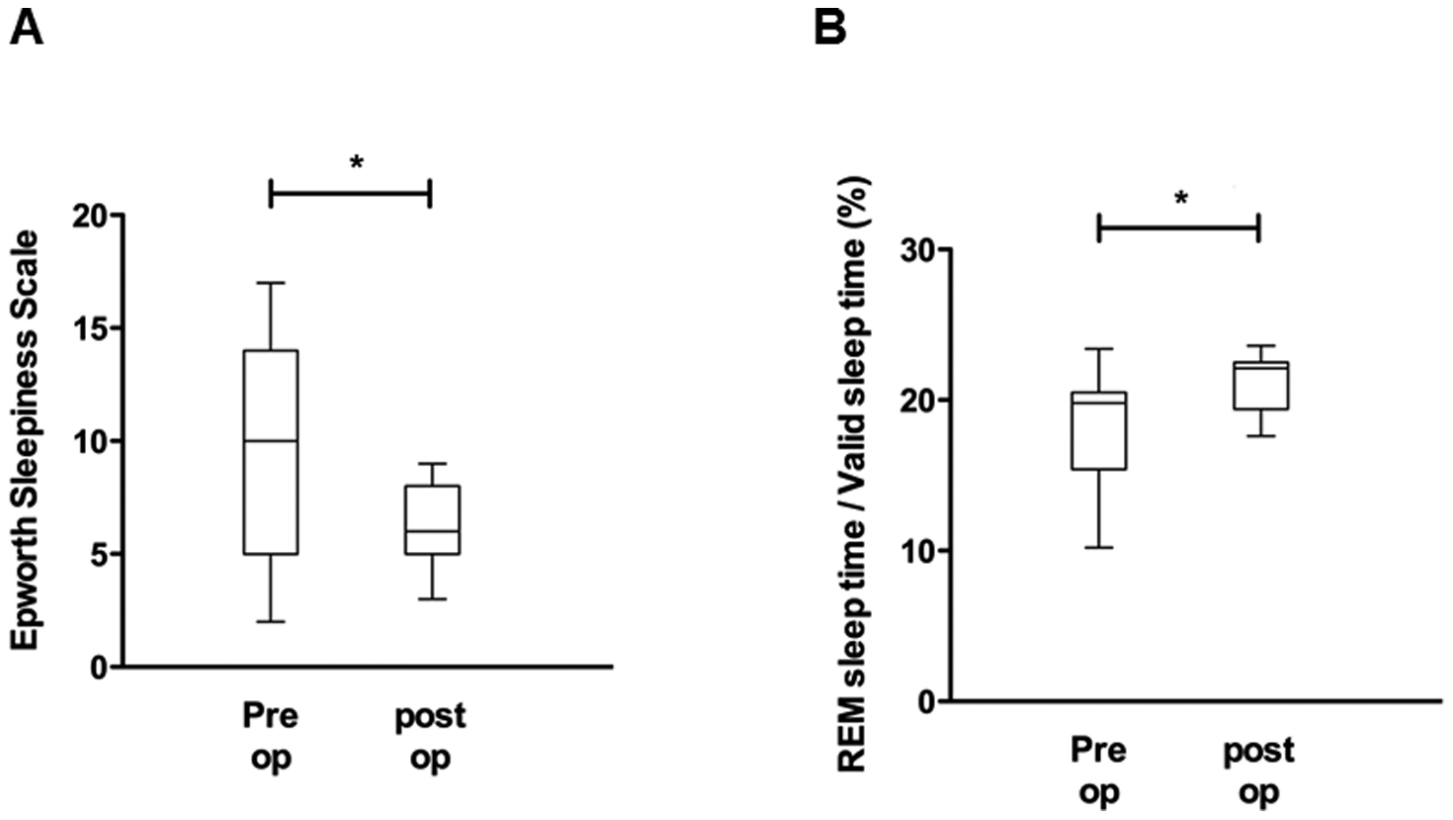
Changes in watch-PAT-derived factors, such as valid sleep time (A) and percentage of REM sleep (B), before and after nasal surgery in the non-responder group (N = 11). ^*^: p<0.05 when comparing the pre-and post-operative values.

Eleven subjects showed less 50% improvement in pRDI (Figure. 6A, 6B) despite significant improvement in MCA (0.41±0.2 cm^2^ to 0.63±0.2 cm^2^) after nasal surgeries. These subjects were classified as non-responder. In terms of OSA severity, two subjects were diagnosed with mild OSA, five patients with moderate OSA, and four patients with severe OSA. Even though there was no significant recovery in the parameters obtained from the watch-PAT, there was significant improvement in the ESS score (10.0±5.1 to 6.4±1.7, Figure. 7A) and the percentage of REM sleep time (17.8±4.3% to 21.3±1.9%, Figure. 7B) suggesting that correction of nasal obstruction contributes to relief of OSA patients' subjective symptoms and facilitates sleep quality.

Seven subjects were recommended for home-based autoPAP (ResMed S9) therpy prior to surgery. They could not tolerate autoPAP pressure and failed autoPAP therapy due to sleep disturbance or poor adherence. These seven patients underwent nasal surgeries for reducing OSA severity but finally were classified in the non-responder group showed significant improvement in nasal breathing after nasal surgeries. Post-operative autoPAP therapy was performed in these subjects and the optimal therapeutic pressure using the autoPAP ranged from 6 to 14 cmH_2_O with mean setting of 8.3 cmH_2_O. The postoperative autoPAP settings decreased six subjects and unchanged in one subject. After one week of post-operative autoPAP therapy, all seven subjects were able to adhere to autoPAP therapy based on a strict definition of adherence (>4 hr/night every day). They maintained autoPAP therapy more than 4 hr/night at least 5 nights a week after nasal surgery.

## Discussion

In the current study, we present evidence that relief of nasal obstruction during sleep and collection of nasal pathologies improved sleep quality and reduced the severity of OSA. In addition, nasal surgeries predictably induced autoPAP tolerability in the subjects who required for postoperative autoPAP due to unresolved OSA.

The main pathologic condition in OSA is airway collapse and many anatomical factors may contribute to this airway collapse. The decrease of nasal patency can promote more negative intraluminal pressure within upper airway and provoke an increase in airway collapse at the level of oropharynx or hypopharynx [15], [16]. Abnormalities of the nose, such as septal deviation, nasal polyps, intranasal benign tumors, inferior turbinate hypertrophy, rhinitis, and even malignancies, may cause or aggravate symptoms of OSA due to severe nasal obstruction and elevated nasal airway resistance [17]. Previous reports have shown that nasal obstruction in normal individuals may lead to an increase in nasal airway resistance, which causes sleep disorder breathing events, including snoring, apnea and hypopnea events [18]–[20]. Therefore, nasal surgeries, such as septoplasty, rhinoplasty, functional endoscopic sinus surgery, and turbinoplasty are occasionally attempted with the goal of altering structural abnormalities and improving nasal patency in OSA subjects.

There is controversy over whether nasal surgery is effective in treating OSA and whether nasal surgery should still be considered a treatment modality for OSA. However some evidence suggests significant improvement in symptoms and sleep study parameters after nasal surgery in OSA patients. Furthermore, nasal surgery results in a positive effect on sleep quality, architecture, position and sleep disordered breathing [4]. This was shown in our study in which nasal surgeries significantly relieved, such as nasal obstruction, snoring and apnea. In addition, we observe significant improvement in sleep parameters, such as pAHI, pRDI, the index for daytime sleepiness, valid sleep time and percentage of REM sleep time after nasal surgery.

Opposing results have been presented in several studies. In one study, nasal surgery alone or in combination with uvulopalatopharyngoplasty showed a limited effect on the severity of OSA or on improvement of sleep quality compared to CPAP treatment [13]. Virkkula et al. suggested that OSA is not relieved by nasal surgery despite improvement in nasal resistance and nasal surgery also does not result in a significant reduction in sleep parameters [21], [22]. These discrepancies may be due to the present of different ethnic groups in the subject population or due to inconsistent evaluation of nasal obstruction in OSA patients during sleep. We assumed that the role of nasal surgery should be addressed from different perspectives and using a more objective evaluation of nasal breathing or fixed nasal obstruction.

We believe that one of most common causes of conflicting results for surgical treatment of OSA is the inaccurate evaluation of anatomical narrowing in OSA subjects. Discrepancies in clinical results after sleep surgery to correct narrowing at the oropharynx or hypopharynx may result from additional narrowing of upper airway. Anatomic factors in the nasal cavity like an enlarged tonsil, macroglossia, redundant pharynx muscles or narrowing at the glottis level can also increase oropharynx and hypopharynx airway resistance. Although airway collapse at the palatal and pharyngeal level would be corrected, snoring, apnea and hypopnea events remain if airway narrowing is still present at the level of the nasal cavity which accounts for about half of total respiratory resistance to airflow. In the present study, we recruited OSA subjects who had fixed airway narrowing at the nose, such as septal deviation and turbinate hypertrophy including retropalatal or retroglossal airway collapse. We estimate that the correction of nasal patency through nasal surgeries after exact evaluation of upper airway contributes to a decrease in the severity of OSA and nasal surgeries that reduce airway resistance might be an effective treatment modality for OSA subjects. Therefore, accurate evaluation of sites of anatomical narrowing including nasal cavity is critical to improve results from surgical treatment of OSA.

We observed several associations between the changes in sleep parameters and patients' subjective symptoms. The 14 responders to nasal surgery exhibited the greatest increases in nasal breathing, along with a significant improvement in OSA severity, valid sleep time and percentage of deep sleep. We concluded that surgical correction for nasal obstruction through septoplasty or turbinoplasty influences upper airway resistance and improves sleep parameters in about 56% of OSA subjects. There were also 11 non-responders who did not show significant improvements in sleep parameters. However, in these subjects, there was an increase in the percentage of REM sleep time and a reduction in daytime sleepiness. This suggests that nasal surgeries, while not always reducing the severity of OSA, may improve sleep quality if fixed nasal pathologies are completely corrected with nasal surgery.

Nasal obstruction or discomfort has been frequently cited as a factor in CPAP intolerance [23]. Many studies have reported a positive effect of nasal surgery on CPAP pressure and compliance. Friedman et al. found a significant reduction in required CPAP pressure levels after nasal surgery in their study involving 40 OSA patients' records [24]. Similarly, Nakata et al. showed a significant decrease in CPAP pressure and improved compliance in 12 subjects [25]. In the present study, we also suggest that nasal surgeries appear to play a positive role in improving autoPAP adherence. Seven subjects who were not able to adhere to autoPAP became more tolerable after their nasal pathologies were corrected and reported improvement in their sleep quality. Based on these findings, we proposed that nasal surgery could be indicated for subjects who cannot tolerate autoPAP, who have a subtherapeutic reduction of their AHI using autoPAP alone, or who may not be able to use a autoPAP machine.

We did not recruit the subjects through a randomized placebo-controlled trial, such as sham surgery. However, symptomatic nasal obstruction was more prominent feature, and perhaps a more prominent aspect of the OSA pathophysiology in those subjects. Therefore only nasal surgeries were performed to the subjects. Although there is also a limitation to our investigation but those patients who chose nasal surgery only would be more likely to have better subjective and objective results with nasal surgery.

In summary, correction of nasal pathologies and relieving nasal patency may improve sleep parameters and reduce subjective symptoms in OSA subjects, which lead to better sleep quality. Although nasal surgery does not always reduce OSA severity, it thought to reliably augment adherence to autoPAP in cases where nasal obstruction is a limiting factor.
